# Photoconductivity Enhancement in Atomically Thin Molybdenum Disulfide through Local Doping from Confined Water

**DOI:** 10.1021/acs.jpcc.3c03442

**Published:** 2023-07-26

**Authors:** Jort D. Verbakel, Annelies Dekker, Harold J. W. Zandvliet, Pantelis Bampoulis

**Affiliations:** Physics of Interfaces and Nanomaterials, MESA^+^ Institute for Nanotechnology, University of Twente, P.O. Box 217, 7500AE Enschede, The Netherlands

## Abstract

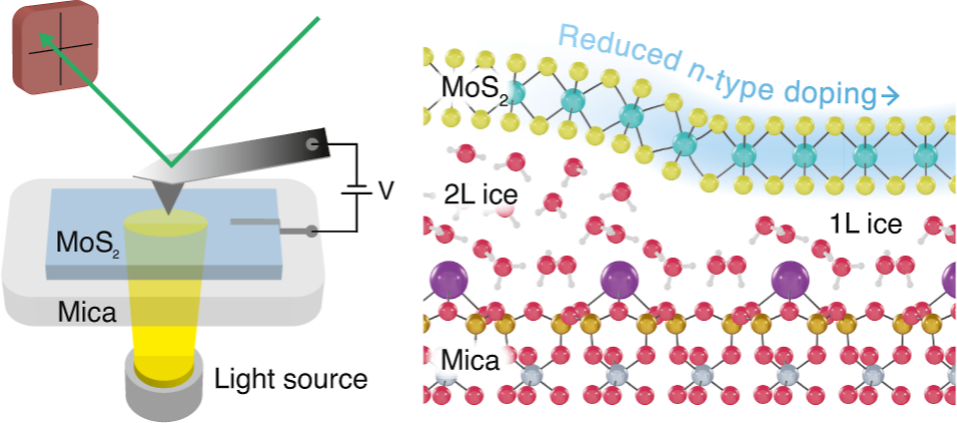

Two-dimensional transition metal dichalcogenide (TMDC) materials have shown great potential for usage in opto-electronic devices, especially down to the regime of a few layers to a single layer. However, at these limits, the material properties can be strongly influenced by the interfaces. By using photoconductive atomic force microscopy, we show a local enhancement of photoconductivity at the nanoscale in bilayer molybdenum disulfide on mica, where water is confined between the TMDC and the substrate. We have found that the structural phase of the water influences the doping level and thus the tunneling barrier at the nanojunction. This leads to an increase in photocurrent and enhanced photopower generation.

## Introduction

One of the many advantages of two-dimensional (2D) materials over conventional bulk semiconductors when it comes to developing future electronics is their significantly lower dielectric screening.^1^ This allows for increased control of the carrier concentration inside these materials, for instance, through chemical dopants or electrostatic gating. A popular class of materials within the family of 2D materials are transition metal dichalcogenides (TMDCs). TMDCs are semiconductors with a large band gap (1–2 eV), which can be converted from indirect to direct by reducing their thickness from bulk to monolayer.^2−4^ This makes them interesting for downscaling devices such as transistors, photodetectors, and photovoltaics.^3,5^ Due to the lower dielectric screening, (opto)electronic devices based on TMDCs are highly sensitive to the interfaces between the TMDC and the substrate, the metallic contacts, and the ambient environment.^6^ While this raises challenges, it also creates opportunities for precise engineering of the TMDC-based devices. Therefore, the choice of substrate and contact materials is vital to the performance of TDMC devices and needs to be thoroughly understood down to the nanoscale.

A popular substrate for TMDC devices in literature has been silicon dioxide (SiO_2_), given its ease of fabrication on standard Si wafers and high crystal quality. However, its relatively high surface roughness and defect density reduce mobilities when using it as a gate dielectric for TMDCs.^6^ Additionally, it is also known to act as a source of hole traps at the interface, altering the photoconductive response of the devices. A compelling alternative dielectric substrate can be found in muscovite mica, a silicate material found in nature. Mica, when cleaved, forms atomically flat terraces,^7−9^ allowing 2D materials to be exfoliated on top, diminishing some of the side effects commonly associated with SiO_2_. However, like SiO_2_, mica is naturally hydrophilic, and upon cleaving the material in open air, water adsorbs onto the surface. When a 2D material is then exfoliated on top of the mica, the water becomes confined: the structure and dynamics of this water have been well established in literature.^10−14^ The structural phase of the water has been found to influence the carriers inside the 2D materials. For instance, Goncher et al. and Bampoulis et al. found that a single water adlayer p-dopes graphene,^11,15^ and van Bremen et al. determined that on tungsten disulfide layers on mica, the structural phase of the water influences the carrier injection mechanism.^16^ The exact origin of these changes in carrier injection, however, remains unclear. Additionally, the opto-electronic properties of TMDCs on mica and the effect of confined molecular species have thus far not received much attention either.

**Figure 1:**
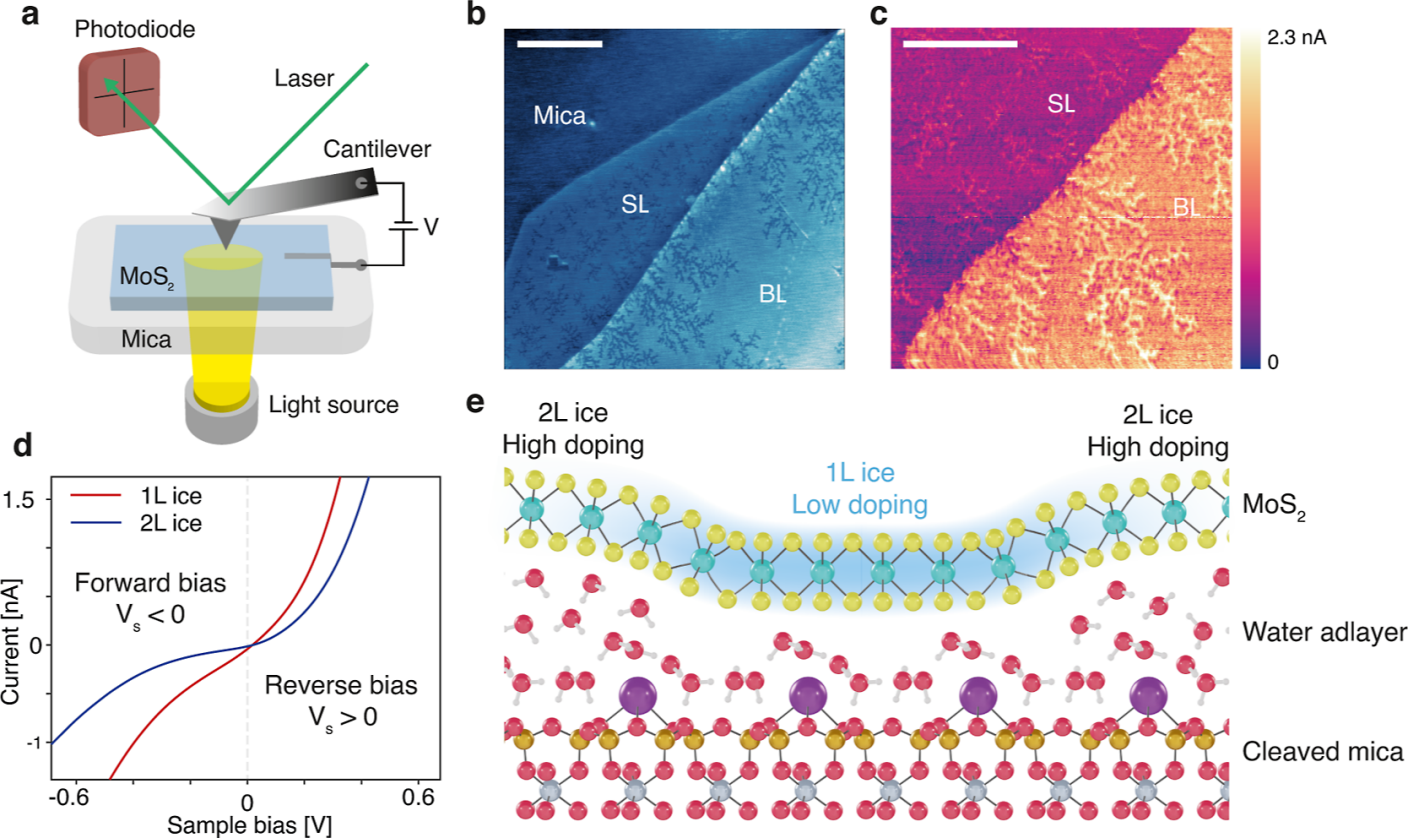
PC-AFM of MoS_2_. (a) Schematic view of the PC-AFM setup. (b) AFM topography image of atomically thin MoS_2_ on mica. Across the single layer (SL) region and bilayer (BL) MoS_2_, ice fractals can be seen. Scale bar: 1 μm. (c) PC-AFM image of a step in the MoS_2_ from SL to BL at a bias of 0.5 V, showing the increased levels of the photocurrent above the regions with 1L ice. Scale bar: 500 nm. (d) Averaged *I*(*V*) curves of BL MoS_2_ above 1L ice (red) and above 2L ice (blue), indicating higher current levels in reverse bias than forward bias due to increased tunneling. (e) Schematic representation of the mos/mica interface, indicating regions that are more strongly n-type doped above the 2L ice, while the region directly above 1L ice has lower doping levels.

In this work, we study the effects of local doping by trapped charges on the photoconductive properties of n-type bilayer (BL) MoS_2_ on mica using photoconductive atomic force microscopy (PC-AFM), as shown in Figure 1a. This method allows us to measure the effects of these charges down to the nanoscale by measuring the current flow between the sample and tip, both in the dark and under illumination. We show that depending on the structure of water trapped between the TMDC and the mica, the doping level of MoS_2_ is changed locally, altering the tunneling barrier that the electrons experience. Additionally, we demonstrate that reducing the water adlayer down to a single ice layer also leads to a significant increase in the photoconductivity and photopower of the MoS_2_ junction.

## Methods

To create samples of MoS_2_ on mica, we mechanically exfoliated crystals of varying thicknesses onto freshly air-cleaved mica substrates. We determined the thickness of the flakes with a combination of AFM and Raman spectroscopy. The sample was placed onto an AFM sample holder with a hole, through which a white LED light source of 3 mW could illuminate the sample. We performed PC AFM measurements by contacting the MoS_2_ flakes with silver paste and applying a bias to the sample, as shown in Figure 1a. We simultaneously measured the topography and current using an Agilent 5100 AFM/STM system with a highly boron-doped diamond tip (AD-2.8-SS, Adama Innovations Ltd.) in contact mode. To reduce the relative humidity of the chamber to induce the growth of the 1L ice crystals, we continuously purged the AFM chamber with N_2_ gas. For details on the sample fabrication details and the Raman identification of the number of layers of MoS_2_, please refer to the Supporting Information (SI).

## Results and Discussion

### Local Doping Effects

When mica is cleaved, a water adlayer adsorbs onto the hydrophilic surface.^17−20^ The cleaving of muscovite mica has been found to preferentially occur at the weak bonds between K^+^ ions and aluminosilicate groups.^21^ We have demonstrated that when reducing the relative humidity, the confined water partially evaporates, leading to the growth of 1L ice crystals with fractal-like appearance.^11^ These ice fractals show up as depressions in AFM topography images, as shown in Figure 1b. The first layer of water forms a hexagonal ice structure over the cleaved mica plane, as first described by Odelius et al.^22^ Unlike the case of water around a free positive ion, the water molecules cover the K^+^ ions with the dipole moment facing away from the ion. The first ice layer then forms a polarized layer, with the more electronegative oxygen facing upward, as shown in Figure 1e. The 2D material directly above the 1L ice then becomes hole-doped. MoS_2_ is usually n-type doped, an effect that is commonly attributed to structural defects.^6,23−25^ Molecular doping of water and oxygen by chemisorption to n-type TMDCs has been reported to reduce electron doping.^1,26^ The presence of 1L ice then leads to a net lower n-type doping. Thicker water adlayers, however, are more liquid-like and do not exhibit a net dipole moment and thus do not dope the 2D material above. When the local conductance of the MoS_2_ layer is measured by using conductive AFM (C-AFM), the 1L ice region shows higher current levels as compared to the 2L ice regions, as shown by Figure 1d. This is remarkable since highly doped semiconductors usually show higher conductivities than more intrinsic ones. As this article will show, this can be attributed to increased tunneling between the tip and sample. If indeed MoS_2_ above the ice crystals is less n-type doped than the surrounding 2L ice regions, the rise in conductivity in these regions is noteworthy. This is because of the nanoscale contact between the AFM tip and the sample, in which the reduced doping level has unusual consequences for the charge transport between the metal and the semiconductor material. In a macroscale metal–semiconductor (M/S) junction, the differences in work function between the materials lead to the formation of the well-known Schottky barrier. Disregarding Fermi level pinning, the Schottky barrier (SB) Φ_B_ is given by the Schottky–Mott relation^1,27,28^
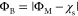
1with Φ_M_ being the work function of the metal and χ_S_ the electron affinity of the semiconductor. At the interface, due to the difference in work function between the metal and semiconductor, a depletion layer forms in tandem with the SB of width *w*, conventionally described by the following relation^28^
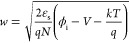
2with ε_s_ being the dielectric constant of the semiconductor, *N* the doping concentration, *V* the applied bias voltage, and ϕ_i_ the built-in potential (ϕ_i_ is in itself dependent on the semiconductor Fermi energy *E*_F,S_, something that is sometimes disregarded). The Schottky barrier height and depletion layer width are critical to understanding the transport mechanisms across the junction. In a conventional device, electrons moving from the metal to the semiconductor are blocked by Φ_B_ and require additional energy to overcome the barrier. In the other direction, electrons only need to overcome ϕ_i_. These conventions usually dictate a forward and reverse bias regime, where before breakdown, the current is higher in forward than in reverse for the same bias. However, when the metal contact size reaches a characteristic length scale, *w* no longer follows the traditional relation and is instead pinned to a size close to the contact radius.^29^ This leads to a shift from thermionic emission-dominated transport to tunneling-dominated transport. This tunneling is most prominent in single to few-layer TMDC thicknesses, as has been experimentally confirmed by C-AFM studies.^16,30^ Consequentially, the rectification properties of the junction change drastically.

**Figure 2:**
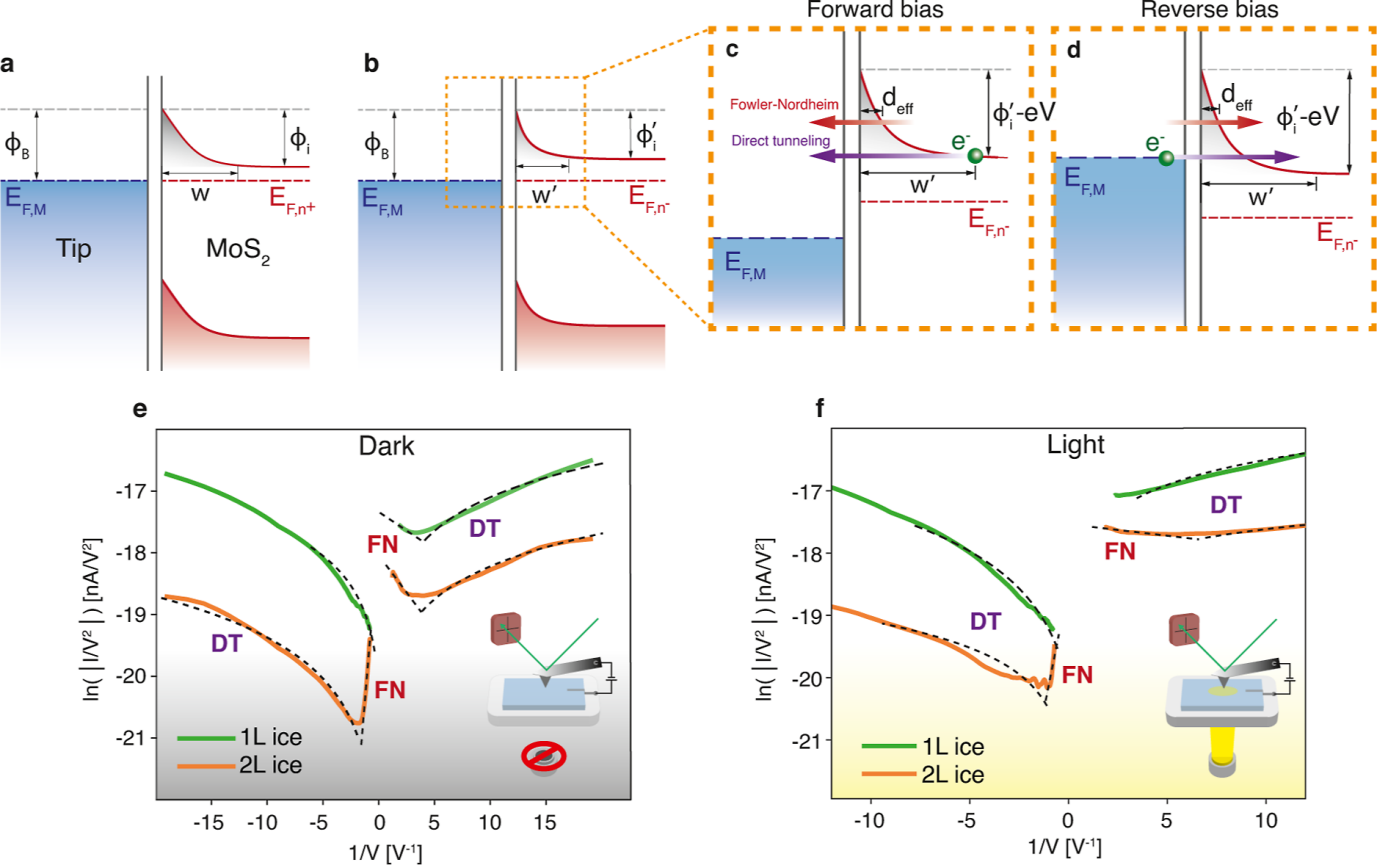
Schematic depiction of the effects of the structure of the underlying water on the metal–semiconductor junction. (a) Zero applied bias above 2L of ice: the heavily doped n-type MoS_2_ (n^+^) forms a depletion layer near the interface with a Schottky barrier height ϕ_B_ and width *w*. (b) Zero applied bias above 1L of ice: the reduced n-type doped MoS_2_ (n^–^) with a reduced barrier width *w*′. (c) Forward bias above 1L of ice: the reduced barrier width *w*′ from the fractal and reduced barrier height ϕ_i_′-eV from the applied bias lead to an increase in the tunneling current. (d) Reverse bias above 1L of ice: The increased band bending due to the applied bias also leads to an increase in the tunneling current, reducing the rectifying behavior of the junction. (e) Fowler–Nordheim (FN) plot, showing an example of curve fitting for FN tunneling and direct tunneling in the dark and (f) with the sample illuminated.

A band diagram of the junction formed by the AFM tip and the n-type doped MoS_2_ is shown in Figure 2a. A Schottky barrier arises, and a depletion layer forms. Note that the size of the depletion layer is extremely small and is of the size of the effective tip contact, extending radially outward due to the 2D nature of the material. When the tip is located above 1L of ice, the reduced doping shifts the Fermi level from the highly doped level *E*_F,n_^+^ to a lower value, indicated by *E*_F,n^–^_, as shown in Figure 2b. Normally, the reduced carrier concentration *N* leads to an increase in *w*; however, due to the nano-sized contact, this extra screening can be neglected,^31^ and *w* does not widen. Instead, due to the shifted Fermi level, ϕ_i_ is decreased, leading to an effective decrease in *w*. When an external bias *V* is applied across the junction, a net current flows. This current flow is dictated by different carrier injection mechanisms, as described in the following section.

### Carrier Injection Mechanism

Carriers can be moved across the junction through three charge injection mechanisms: thermionic emission, direct (DT) tunneling, and Fowler–Nordheim (FN) tunneling. In bulk semiconductors, the wide depletion region makes thermionic emission the dominant charge injection mechanism; in small contacts, tunneling becomes much more prominent due to the electrons being able to tunnel through the small depletion layer. The electrons can tunnel through a barrier smaller than *w*, denoted by an effective barrier width *d*_eff_. In Figure 2c,d the tunneling mechanisms are indicated both in the forward bias and reverse bias regimes. Depending on the carrier injection mechanism and the bias regime, electrons feel a different combination of barrier height (usually proportional to Φ_*B*_) and *d*_eff_. In case of the MoS_2_ above 1L ice, the lower position of *E*_F_ leads to a relatively lower tunnel barrier for both mechanisms. This effect is amplified in reverse bias since the band bending is stronger. This then leads to lower tunneling barriers in reverse bias than in forward bias, which leads to higher currents in reverse bias: an effective reverse of the rectification of the junction. The van der Waals gap (typically about 3 Å) between the sample and tip poses an additional tunneling barrier; however, by keeping the contact force exerted by the tip on the sample constant throughout measurement, the van der Waals barrier width and height remain constant.

The carrier injection mechanism can be determined by analyzing the shape of *I*(*V*) characteristics of the junction. Previous PC-AFM studies of TMDC samples on indium–tin–oxide have shown this by current imaging at different sample biases.^30,32^ However, this limits the resolution in the voltage regime significantly. By doing *I*(*V*)-grid spectroscopy instead, the mechanism can be studied with higher resolution in the voltage regime while allowing spatial resolution at the nanoscale. We studied the presence of thermionic emission, direct tunneling, and FN tunneling by making three types of fits of the same *I*(*V*)-curve at every grid point, employing a method similar to that used by ref (16). We describe direct tunneling by^1,16^
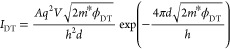
3with *A* being the effective contact area, *q* the elementary charge, m* the effective mass of the electron, *d* the tunnel barrier width, and *h* the Planck constant. We describe FN tunneling by^1,16,30,32^
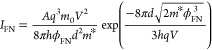
4By plotting ln(*I*/*V*^2^) as a function of ln(|1/*V*|) for direct tunneling and ln(*I*/*V*^2^) as a function of 1/*V* for FN tunneling, we can make linear fits to determine the tunneling parameters  and *d*ϕ_FN_^3/2^. Figure 2e,f shows the fitting of an *I*(*V*) curve for both the dark and illuminated case. At lower currents, DT dominates, and at higher current levels, FN tunneling takes over. However, as shown in the figure, when illuminating the sample, DT is pushed toward higher currents, and FN can disappear altogether due to the upper current limit of the *I*–*V* converter at 10 nA.

**Figure 3:**
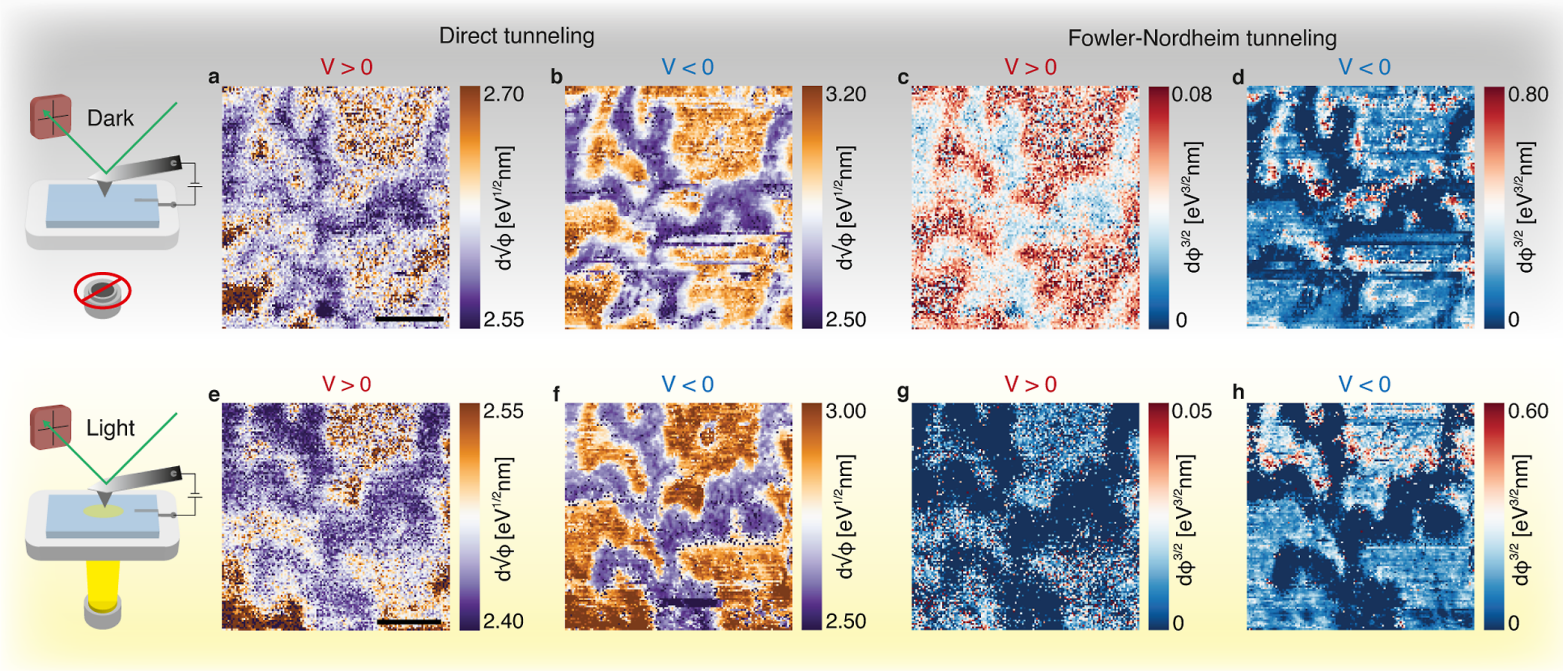
Spatial distribution of the tunneling parameters,  and *d*ϕ_FN_^3/2^, in the vicinity of a 1L ice fractal, in the dark (a–d) and under illumination (e–h). A zero parameter indicates the absence of the carrier injection mechanism. Scale bars: 50 nm. (a) Direct tunneling, RB, dark. (b) Direct tunneling, FB, dark. (c) FN tunneling, RB, dark. (d) FN tunneling, FB, dark. (e) Direct tunneling, RB, light. (f) Direct tunneling, FB, light. (g) FN tunneling, RB, light. (h) FN tunneling, FB, light.

In our measurements, we have also fitted for thermionic emission. However, on BL MoS_2_,  in none of the *I*(*V*)-curves measured, a proper TE fit could be established. This is likely due to the relatively high Φ_B_ as compared to the tunneling barriers. We found that on six layer thick MoS_2_, however, TE is present along with FN tunneling, which is consistent with similar measurements on WS_2_, where TE was only measured in multilayer samples.^16^Figure 3 shows high-resolution maps of the extracted barrier parameters,  and *d*ϕ_FN_^3/2^ , both in the dark and when the sample is illuminated. We distinguish between the FB regime (*V*_sample_ < 0) and the RB regime (*V*_sample_ > 0). There is a clear contrast between the 1L ice region and the 2L ice regions. First, the DT parameter is lower on the 1L region for both bias regimes. Additionally, we observe a generally lower DT parameter in reverse bias as compared to that in forward bias; this is likely due to the lowered Fermi level in reverse bias, which makes the effective tunnel barrier width *d*_eff_ lower compared to that in forward bias. These results match the observation that the forward bias is defined as *V*_sample_ < 0, where we actually observe lower currents with respect to *V*_sample_ > 0, which corresponds to reverse bias for a metal/n-type semiconductor junction. To avoid further confusion, from this point onward, we will refer to forward bias with the sample bias *V* < 0 and to reverse bias with *V* > 0.

When comparing the FN parameters, we see that for positive bias voltages, the 1L ice region shows lower values as compared to the 2L water region and that positive bias shows lower values than negative bias. However, at negative bias, FN tunneling is not observed on the 1L at all. This can be explained by two possible effects. First, the current is already too high at lower bias voltages, saturating the *I*–*V* converter, preventing us from observing FN tunneling. Second, it could be that the triangular barrier disappears due to minimal band bending near the flat-band condition.^33^ In this case, direct tunneling is still present through the van der Waals gap between the sample and tip.

When we turn on the light, we observe a similar trend, except that in the positive bias regime, we no longer observe FN tunneling on the 1L region. Most likely, this can be attributed to the first argument mentioned above, i.e., the increase in current pushes the FN regime outside of our measurement window. Otherwise, the carrier injection mechanisms do not differ significantly from the dark current case. However, we do observe two general trends in the tunneling parameters. First,  is lower under illumination regardless of the water phase and bias, while on 2L ice, *d*ϕ_FN_^3/2^ is lower at positive bias and approximately equal at negative bias.

### Photocurrent Generation

**Figure 4:**
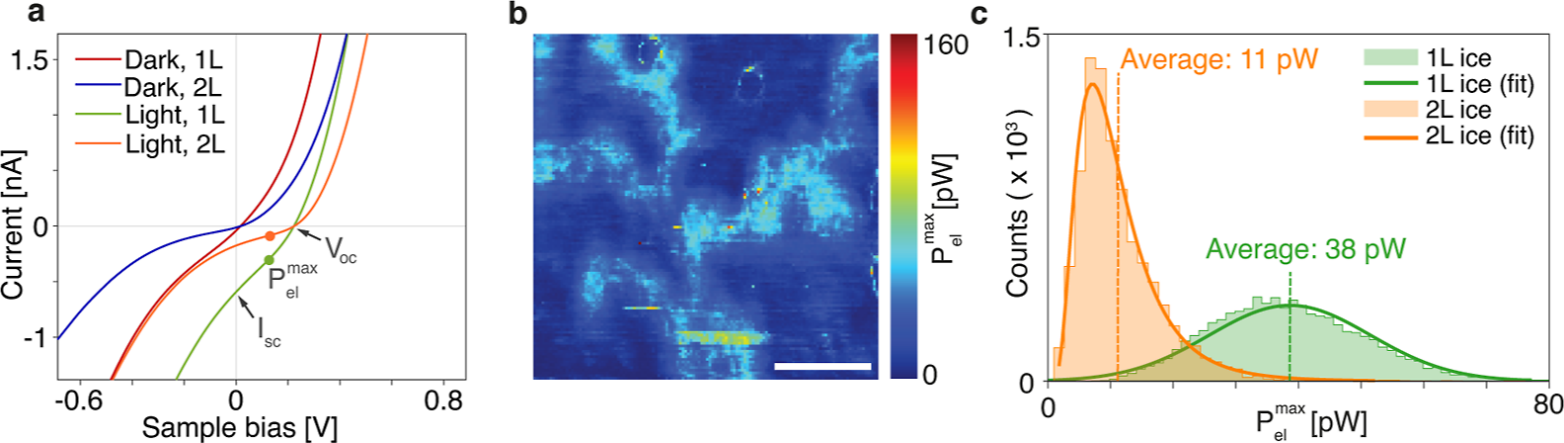
Photopower generation in the metal–MoS_2_ nanojunction. (a) Average *I*(*V*)-curves for the four different conditions. (b) *P*_el_^max^ plotted when illuminating the sample, showing significantly higher photopower for the 1L ice region as compared to that for the 2L ice region. Scale bar: 50 nm. (c) Histogram showing the distribution of *P*_el_^max^ across the measured area.

The *I*(*V*)-grid spectroscopy allows us to locally extract not only the carrier injection mechanism with and without illumination but also the features in the photocurrent. As shown in Figure 4a, by turning on the light, the *I*(*V*)-characteristics differ quite substantially. By illuminating the sample, photocurrent is generated by creating electron–hole pairs in MoS_2_. Even at zero applied bias, a sizable current *I*_sc_ (short-circuit current) is generated. Only an applied *V*_oc_ (open-loop bias) can cancel the spontaneously generated photocurrent; this is described as the photovoltaic (PV) effect.^5^ In a conventional metal/n-type Schottky junction, the photoexcited carriers are naturally pushed away from the junction by the built-in electric field, and *V*_oc_ is then in the forward bias regime. However, like before, we observe an opposite effect in the nanojunction case, where *V*_oc_ is actually in reverse bias. This effect is likely due to the depletion region being of the same size as the contact: the depletion region is where charge separation of the photoexcited carriers occurs,^34^ which means that the photoexcited carriers have very little chance to escape and are immediately collected by the tip.

For every *I*(*V*)-curve, there is a maximum power *P*_el_^max^ that is generated in the fourth quadrant (*V* > 0, *I* < 0). We can plot this value for each point on the grid in Figure 4b, showing the differences for 1L and 2L ice. We see a sharp increase in the generated photopower with the decreased doping, with an average of 39 pW for the 1L ice region and 11 pW for the 2L ice region. The distributions of the photopower values across the measured area are shown in Figure 4c. The 1L region shows a distribution close to a normal distribution, while the 2L region is more close to a lognormal distribution. Increased n-type doping has been experimentally tied to reduced optical adsorbance^35^ and decreased n-type doping with an enhanced photoabsorption.^36,37^ Therefore, a likely reason for the increase in photocurrent on the 1L ice region is the increased creation of photoexcited carriers due to the local decrease in doping level. Photogating is another possible effect, where the defects in MoS_2_ can lead to an increased photocurrent due the trapping of photoexcited holes, locally decreasing the conduction band minimum, increasing the electron current under illumination. Additionally, trapped water underneath MoS_2_ has been proposed as a source of the photovoltaic/photogating effect, where the water confined between MoS_2_ and SiO_2_ acts as charge trap.^38^ Given the specific ordering of the water molecules on top of the mica, this is hard to compare to the SiO_2_ case. However, we cannot completely rule out the photogating effect since this requires a variable back-gate voltage or a chopped light source to properly distinguish photogating effects from photoconductive effects.^5,38^ In Figure 4b, a small defect can be identified on the 2L ice region, which shows up as a ring-like structure in the photopower plot. Here, the increase in photocurrent can be tied to a very local photogating effect, showing an increased photopower comparable to the 1L ice. On six-layer MoS_2_, however, the defects dominate the photocurrent and photopower. We attribute this to additional MoS_2_ layers screening the effects of the confined water below. For more details, please refer to the Supporting Information.

### Potassium Vacancies

**Figure 5:**
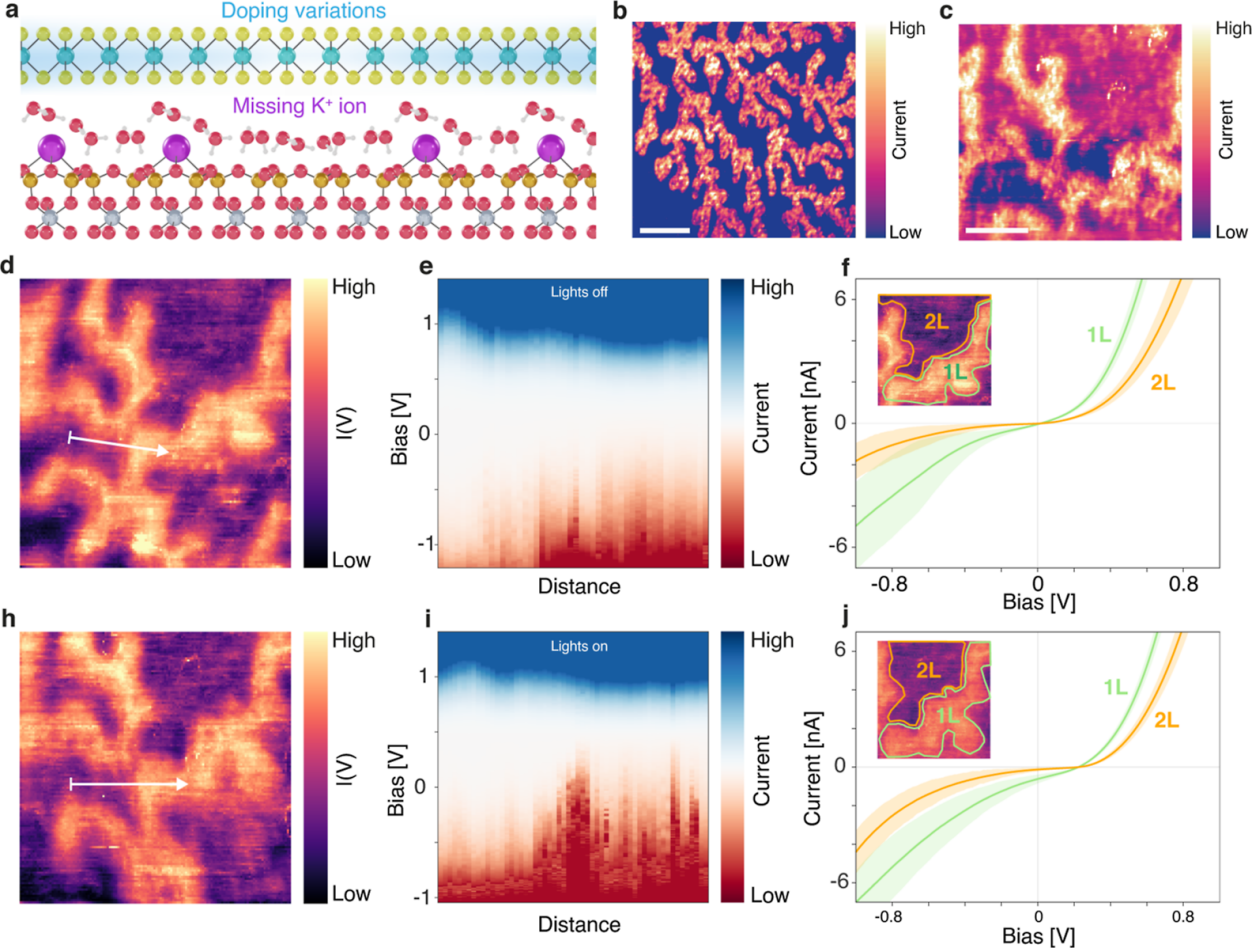
Changes in the photocurrent due to missing potassium ions. (a) Schematic representing the local changes in the ordering of water molecules when the potassium ions are locally absent, which causes variation in the local doping on the 1L ice region. (b) Photocurrent map on six layer MoS_2_ at 0.7 V bias. Scale bar: 200 nm. (c) Photocurrent map on BL MoS_2_ at −0.5 V bias. Scale bar: 50 nm. (d,e) Line *I*(*V*) profile in the dark, taken at the location indicated by the white arrow in (d). (f) Variations in the *I*(*V*) curves for 1L ice and 2L ice in the dark, showing the average curves and their standard deviation. Inset: selected area for averaging the curves. (h,i) Line *I*(*V*) profile with light along the same direction as (d). (j) Variations in the *I*(*V*) curves for 1L ice and 2L ice with light.

When mica is cleaved, it preferably separates where the aluminosilicate layers are bonded to the potassium ions in between, since those bonds are relatively weak.^21,22,39^ To retain electronic neutrality, half of the K^+^ ions remain on the mica substrate after cleaving.^8,39,40^ We have previously demonstrated that due to presence of the structured ice layer, the distribution of these ions is non-uniform.^18^ In this study, we show that the regions where the potassium is missing correlate to a higher conductance in graphene due to increased p-type carrier density. Figure 5a shows the effect of the K^+^ vacancies on MoS_2_: due to the missing ions, MoS_2_ is affected not only by the polarized ice but also by the negatively charged aluminosilicate groups in the mica, reducing the doping level locally inside the 1L ice region even further. This causes fluctuations in the tunneling current, which is most noticeable inside the 1L ice regions. Figure 5b,c shows this effect for six-layer and BL MoS_2_,  respectively, where strong current variations are present inside the 1L ice region. Additionally, we show these current fluctuations with line *I*(*V*) spectroscopy in Figure 5d–f. The fluctuations are significantly stronger in the negative sample bias regime. This can be explained by changes in the direct tunneling barrier. In the negative bias regime (FB), electrons experience a barrier defined by the built-in potential ϕ_*i*_ and the width *w*′, as shown in Figure 2c. In the positive bias regime (RB), however, the barrier is defined by the Schottky barrier Φ_B_, and the width *w*′. Φ_B_ is constant in the absence of defects, while ϕ_i_ is dependent on the Fermi level and therefore the local doping level. Due to the changes in ϕ_i_, it is, therefore, to be expected that the tunneling current is much more sensitive to the K^+^ distribution in the negative bias regime.

## Conclusions

Using PC-AFM, we have shown that the phase of water trapped between mica and BL MoS_2_ not only changes the tunneling of electrons between the tip and MoS_2_ but also significantly influences the photocurrent. Our high-resolution photocurrent maps and *I*(*V*) grid measurements show that the phase of the water and the varying potassium distribution of the mica change the doping level down to the nanoscale. Due to the small size of the contact and the 2D nature of the material, the depletion layer is not widened by the reduction in carrier density, and the width and height of the tunnel barriers are closely tied to the Fermi level of MoS_2_. We have found that the charge injection mechanism is tunneling, and where the water is in a polarized 1L ice state, the reduced n-type doping leads to even lower tunneling barriers for the charge carriers. For MoS_2_ regions over 2L of water, this polarization is significantly reduced, and the substrate recovers its stronger n-type character. When the sample is illuminated with white light, the tunnel barriers  for direct tunneling and *d*ϕ_FN_^3/2^ for FN tunneling reduce even further, and an enhanced photocurrent and photopower are measured. We attribute this effect to an increased generation of photoexcited carriers due to the lower doping levels, and carriers can efficiently be transferred between the semiconductor and the tip due to the low tunnel barrier.

## Supporting Information Available

Additional sample fabrication details, determination of the amount of MoS_2_ layers by AFM and Raman spectroscopy, and details about *I*(*V*) curve fitting and measurements on 6L MoS_2_ (PDF)
