# Supplementary material for: Photoconductivity Enhancement in Atomically Thin Molybdenum Disulfide through Local Doping from Confined Water
Source: J Phys Chem C Nanomater Interfaces. 2023 Jul 26;127(34):17171–8. doi: 10.1021/acs.jpcc.3c03442 (PMC10476179; doi:10.1021/acs.jpcc.3c03442)
Supplement: Supplementary file 1 — jp3c03442_si_001.pdf [file jp3c03442_si_001.pdf]

# SUPPLEMENTARY INFORMATION

# Photoconductivity Enhancement In Atomically Thin Molybdenum Disulfide Through Local Doping From Confined Water

Jort D. Verbakel,[†,‡] Annelies Dekker,[†,‡] Harold J.W. Zandvliet,[†] and Pantelis Bampoulis[*,†]

†*Physics of Interfaces and Nanomaterials, MESA*$^{+}$ *Institute for Nanotechnology, University of Twente, P.O. Box 217, 7500AE, Enschede, The Netherlands*

‡*Both authors contributed equally to this work.*

E-mail: p.bampoulis@utwente.nl

# Contents


**1 Sample fabrication details** **S3**

**2 Layer number determination** **S3**

2.1 Confocal Raman spectroscopy . . . . . . . . . . . . . . . . . . . . . . . . . . S4

2.2 AFM step height measurement . . . . . . . . . . . . . . . . . . . . . . . . . S6

**3 Fitting of carrier injection mechanism** **S8**

3.1 Thermionic emission . . . . . . . . . . . . . . . . . . . . . . . . . . . . . . . S8

3.2 Direct tunneling . . . . . . . . . . . . . . . . . . . . . . . . . . . . . . . . . S10

3.3 Fowler-Nordheim tunneling . . . . . . . . . . . . . . . . . . . . . . . . . . . S10

3.4 Photocurrent measurements few-layer $MoS_2$ . . . . . . . . . . . . . . . . . . . . S1

# 1 Sample fabrication details

The sample fabrication is explained in figure S1 below.

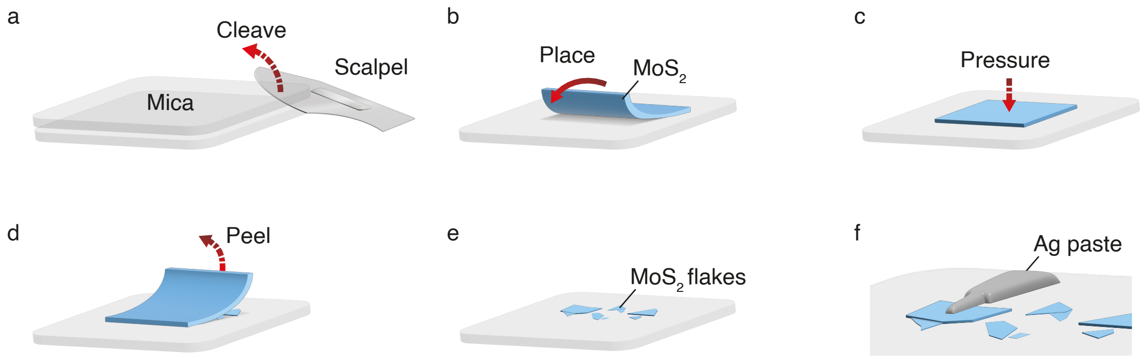


Figure S1: Detailed description of sample fabrication. (a). The mica is cut into small squares of about 1.5×1.5 cm. A scalpel is inserted between two layers of mica and the scalpel lifts off the top part, revealing a flat, clean mica surface. This is all done in ambient atmosphere, such that the mica surface is immediately covered with a thin water layer. (b) $MoS_2$ is freshly exfoliated from 3M Magic scotch tape until large area flakes (a few mm in size) can be cleaved away and picked up from the tape with sharp tweezers. The flake is then carefully laid on top of the mica. (c) The flake is then pressed against the mica to ensure conform contact, but not to shatter the flake. (d,e) Next, the flake is peeled off the mica, leaving behind small $MoS_2$ flakes with varying thicknesses. (f) When a flake with atomically thin parts is identified with optical microscopy and Raman spectroscopy, the flake is contacted with Ag paste to allow for PC-AFM measurements.

# 2 Layer number determination

To identify the amount of layers present on the $MoS_2$ sample, atomic force microscopy can be of great value, but cannot reliablly identify the amount just by itself. This is caused by the different sample-tip interactions between the mica surface and the $MoS_2$ which can cause the step height to yield misleading values. However, in combination with confocal Raman spectroscopy, this becomes much more reliable. In the following sections, the layer number identification process will be explained in further detail.

## 2.1 Confocal Raman spectroscopy

Raman mapping was performed using a WiTec alpha300 R confocal Raman microscopy system and analyzed using a principal component analysis (PCA) algorithm.[1] The Raman excitation wavelength was 532 nm at a laser power of 500 μW using grating of 600 lines/mm. The approximate region of the flake that was analyzed is shown in figure S2(a,b). The minimum pixel size of the Raman mapping mode was 500×500 nm, which is too large to individually visualize the single layer part of the flake, but is enough to visualize and map the bilayer part. In combination with AFM measurements, we could then determine the thinnest part measured with the AFM to be monolayer $MoS_2$.

As shown in figure S2(c,d), the distance between the $E_{2g}$ and $A_{1g}$ peaks is 24.6 $cm^{-1}$, which corresponds to 5-6 layer $MoS_2$.[2–4] The changes in frequency difference between 5 layers or more become significantly smaller than for lower amounts of layers, making it difficult to distinguish by Raman spectroscopy alone. However, the value we find here is closer to 5 layers than to 6 layers. On the other hand, the measurement shown in in figure S2(e,f) clearly indicates bilayer $MoS_2$ with a peak location difference of 21.8 $cm^{-1}$. Given that in the AFM data of figure S3a and b we observe a step height of 0.68 nm, this corresponds to a step from monolayer to bilayer $MoS_2$ and not with a step from bilayer to 5-6 layers. Therefore we conclude that at the very end of the flake a small region of monolayer $MoS_2$ is present, and the flake goes from monolayer to bilayer to six layers to bulk.

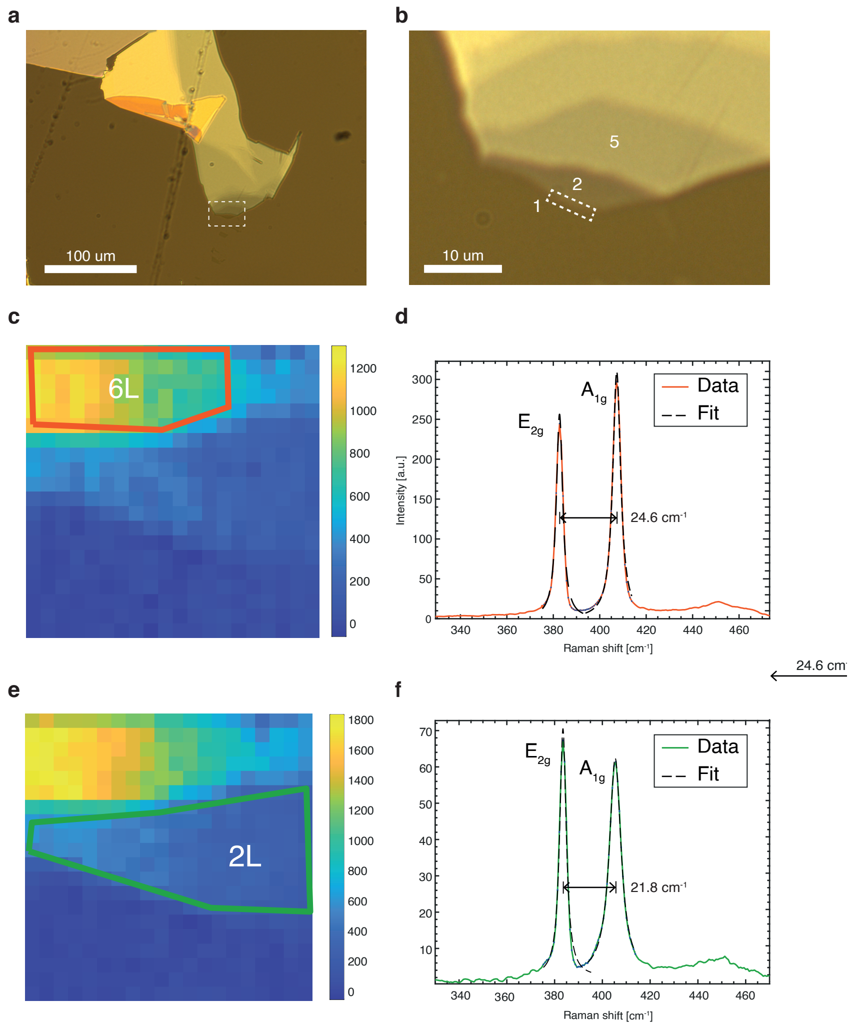


Figure S2: Optical and confocal Raman microscopy images of the studied flake. (a) Optical microscopy image of the $MoS_2$ flake, showing the bulk and atomically thin parts (white square). (b) Higher resolution image of the outlined part in (a). The single layer $MoS_2$ is not visible due to the low optical contrast on the mica. (c) Raman intensity map of the intensity of the $E_{2g}$ peak, indicating the 5-layer thick part of the flake. The (d) Averaged spectrum of the red outlined region in (c). The $E_{2g}$ peak is located at 383.5 cm$^{-1}$ with a FWHM of 3.3 cm$^{-1}$, and the $A_{1g}$ peak is located at 405.4 cm$^{-1}$ with a FWHM of 5.9 cm$^{-1}$. (e) Raman intenisty map of the $E_{2g}$ peak, indicating the 2-layer thick part of the flake. (f) Averaged spectrum of the red outline region in (e). The $E_{2g}$ peak is located at 382.7 cm$^{-1}$ with a FWHM of 3.3 cm$^{-1}$, and the $A_{1g}$ peak is located at 407.4 cm$^{-1}$ with a FWHM of 4.1 cm$^{-1}$.

## 2.2 AFM step height measurement

AFM topographies of the $MoS_2$ flake on which the PC-AFM experiments were performed are shown. In figure S3(b), a height profile across the mica-1L-2L part is shown. As argued before, the step height of mica to the $MoS_2$ is around half of the expected value for a single layer, and cannot be relied upon due to the difference in mica-tip interactions and $MoS_2$-tip interactions. However, the second step is about 0.68 nm, which corresponds to a step of a single layer of $MoS_2$.[5] In figure S3(c), a line profile at a different location of the flake is shown. The top left part is at the same level as the lower right part of figure S3(a). A transition to few-layer $MoS_2$ is shown, with a difference of 3.16 nm between the highest and lowest parts. Taking a monolayer step height to be 0.68 nm as found before, this corresponds to  4.6 layers. This, taken together with the Raman spectrum for the few-layer $MoS_2$ part, makes it most likely 6 layers instead of 5.

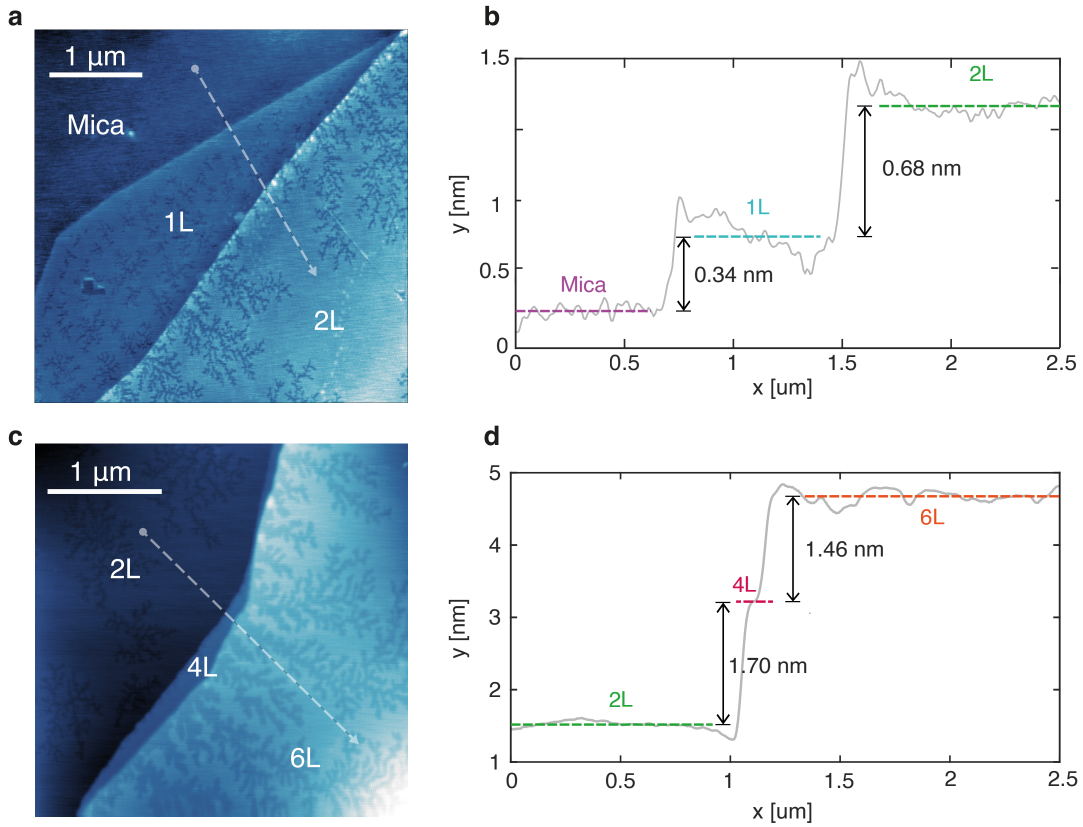


Figure S3: Optical and confocal Raman microscopy images of the studied flake. (a) AFM topography image (also shown in main text) showing the mica, 1L and 2L $MoS_2$. (b) Line profile cross-section of the path shown in (a), incidicating the different step heights. (c) AFM topography image of a different region of the same flake as (a), but now showing the 2L and 6L region with a very narrow region of 4L in between. (d) Line profile cross-section of the path shown in (c), indicating the different step heights.

# 3 Fitting of carrier injection mechanism

## 3.1 Thermionic emission

For thermionice emission, we used the following model:

$$I_{\mathrm{TE}} = I_0 \exp\left(\frac{qV}{\eta k_B T}\right)\left[1 - \exp\left(\frac{-qV}{k_B T}\right)\right] \tag{1}$$

with $\eta$ the ideality factor. Furthermore:

$$I_0 = AA^* T^2 \exp\left(\frac{q\Phi_B}{k_B T}\right) \tag{2}$$

with $\Phi_B$ the Schottky barrier height, $A$ the effective tip contact radius and $A^*$ the Richardson constant ($A^* = 4\pi q m^* k_B^2/h^3$), where $m^*$ is the effective mass of an electron. Assuming $qV > k_B T$, and linearizing by plotting $\ln(I)$ as a function of $V$:

$$\ln(I) = \ln\left(AA^* T^2\right) + \frac{q\Phi_B}{k_B T} - \frac{q}{\eta k_B T} V \tag{3}$$

The effective contact radius can be approximated using the Hertz equation for a contact between a sphere and an elastic half-space:

$$r^3 = \frac{3FR}{4}\left(\frac{1-\nu_t^2}{E_t} + \frac{1-\nu_s^2}{E_s}\right) \tag{4}$$

with $r$ the contact radius between tip and sample, $F$ the applied force, $R$ the radius of the tip, $v\nu_t$ (0.20) and $\nu_s$ (0.22) the Poisson's ratio of the tip and sample respecitvely, and $E_t$ (1000 GPa) and $E_s$ (270 GPa for $MoS_2$) the elastic moduli of the sample and tip. $A$ is then given by $A = \pi r^2$. We determine $F$ by analyzing force-distance to find the deflection sensitivity to determine the force as a function of contact setpoint. For the cantilever used in our measurements, we find $F$ to be between 10 and 40 nN. This results in a value for $r$

between 0.5-1 nm.

Using the TE model, we found that the linear regime of eq. 3 was only in the small-bias regime, where the assumption $qV > k_BT$ at room temperature does not hold very well. Only a few data points were established per fit, making values for $\Phi_B$ and $\eta$ unreliable. Therefore, we conclude that TE is not contributing as a carrier injection mechanism in the M/S nanojunction for the BL $MoS_2$. However, measurements on 6L $MoS_2$ showed consistent TE at low bias regimes, as figure S4 shows. We see that the 1L ice region shows lower ideality factors, and slightly lower Schottky barrier values. However, the Schottky barrier is altered much more drastically by the presence of defects in the $MoS_2$, lowering $\Phi_B$ to as low as 0.16 eV. This indicates that with increasing layer number, thermionic emission becomes increasingly present as a charge injection mechanism. This is consistent with similar measurements performed on $WS_2$ on mica.[6]

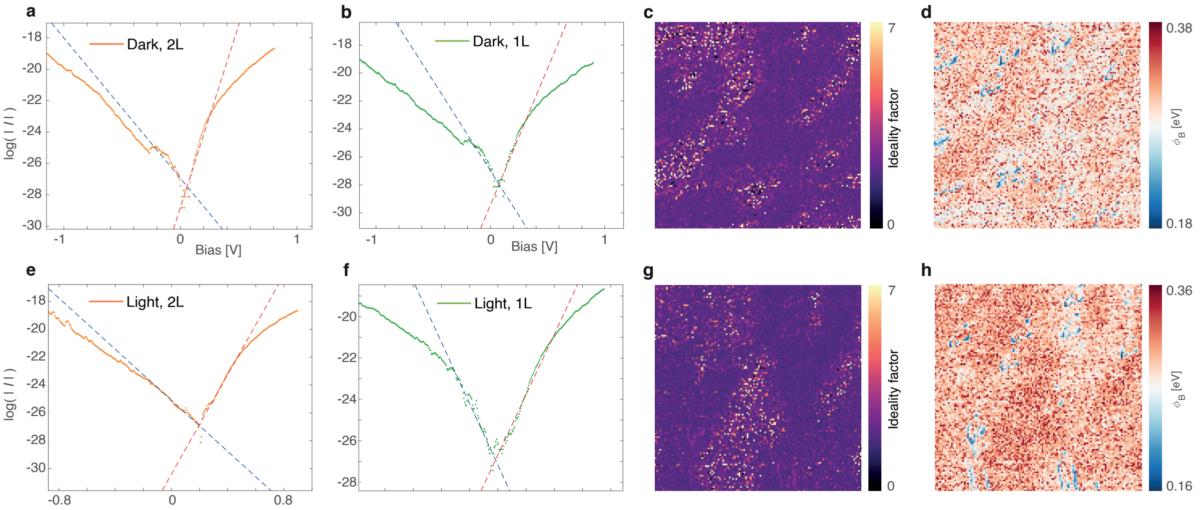


Figure S4: Thermionic emssion in 6L $MoS_2$ in the dark (a-d) and illuminated (e-h). (a,b) TE fit of a single $I(V)$ curve on 2L ice and 1L ice, respectively. (c,d) The locally extracted ideality factor $\eta$ and Schottky barrier $\Phi_B$ respectively, showing contrast between 1L and 2L ice regions. (e,f) TE fit of a single $I(V)$ curve on 2L ice and 1L ice, respectively. (g,h) The locally extracted ideality factor $\eta$ and Schottky barrier $\Phi_B$ respectively, showing contrast between 1L and 2L ice regions.

## 3.2 Direct tunneling

In the case of direct tunneling, equation (3) in the main text can be linearized using the following relation:

$$\ln\left(\frac{I}{V^2}\right) = \ln\left(\frac{Aq^2\sqrt{2m^*\Phi_B}}{Vh^2d}\right) - \frac{4\pi d\sqrt{2m^*\Phi_B}}{h} \tag{5}$$

with $h$ the Planck constant and $d$ the effective barrier width. In this linear relation, the barrier parameter $d\sqrt{\Phi_{\mathrm{DT}}}$ can be determined. As mentioned in the main text, we plot $\ln(I/V^2)$ as a function of the absolute value of $I/V$. From the $y$-intercept (right term in equation 5) we extract $\Phi_{\mathrm{DT}}$.

## 3.3 Fowler-Nordheim tunneling

For Fowler-Nordheim tunneling we use:

$$\ln\left(\frac{I}{V^2}\right) = \ln\left(\frac{Aq^3m_0}{8\pi h\Phi_B d^2 m^*}\right) - \frac{8\pi\sqrt{2m^*}\Phi_B^{3/2}d}{3hqV} \tag{6}$$

with $m_0$ the electron rest mass. We then plot $\ln(I/V^2)$ as a function of $1/V$, and from the slope of the graph (right term in equation 6) we extract the barrier parameter $d\Phi_{\mathrm{FN}}^{3/2}$.

## 3.4 Photocurrent measurements few-layer $MoS_2$

Figure S5 shows measurements on the 6L thick part of the $MoS_2$ flake. As figure S5a and b show, the ice layer thickness still influneces the current levels, albeit much less so than in the case of 2L $MoS_2$. This can be attributed to the increased screening from the extra layers. Instead, defects in the $MoS_2$ show the highest current levels, dominating the dark and illuminated case. In the photopower plot of figure S5c, the ice fractals do not provide any significant contribution to the photopower. This is also shown in the photopower distribution of figure S5e. Instead, the defects are spots of highly increased photopower.

It is also clear from figure S5d that with the thicker $MoS_2$, the increase in current from the 1L ice is significantly less prominent than in the bilayer case. This is consistent with the strongly increased contribution of thermionic emission to the current, as shown in section SI 3.1. Due to the reduced contribution of tunneling, the effects of the reduced tunneling barrier by the 1L ice disappear. This is most evident at negative sample biases.

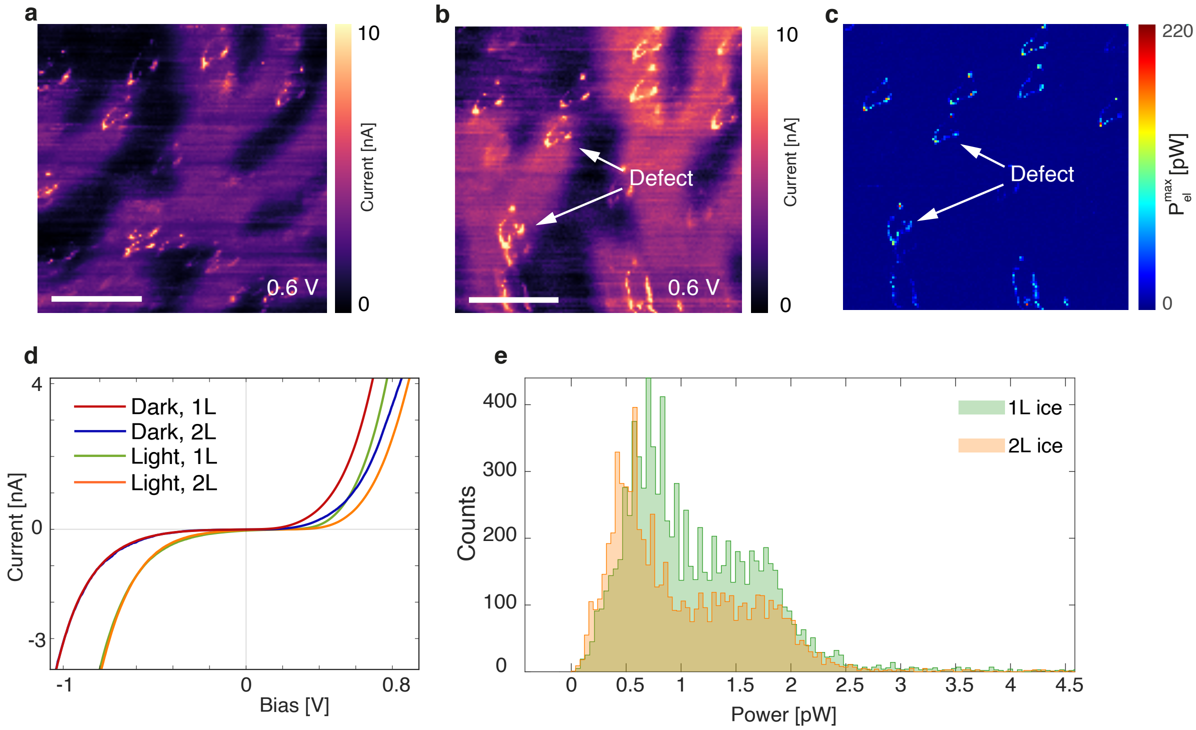


Figure S5: Photocurrent in 6L $MoS_2$(a) $I(V)$ map at 0.6 V in the dark. (b) $I(V)$ map at the same sample bias, when illuminated. (c,d) The locally extracted ideality factor $\eta$ and Schottky barrier $\Phi_B$ respectively, showing contrast between 1L and 2L ice regions. (e,f) TE fit of a single $I(V)$ curve on 2L ice and 1L ice, respectively. (g,h) The locally extracted ideality factor $\eta$ and Schottky barrier $\Phi_B$ respectively, showing contrast between 1L and 2L ice regions.

# References


1. Nair, S.; Gao, J.; Yao, Q.; Duits, M. H. G.; Otto, C.; Mugele, F. Algorithm-improved high-speed and non-invasive confocal Raman imaging of 2D materials. National Science Review **2020**, 7, 620–628.

2. Lee, C.; Yan, H.; Brus, L. E.; Heinz, T. F.; Hone, J.; Ryu, S. Anomalous lattice vibrations of single- and few-layer $MoS_2$. ACS Nano **2010**, 4, 2695–2700.

3. Papanai, G. S.; Sharma, I.; Kedawat, G.; Gupta, B. K. Qualitative Analysis of Mechanically Exfoliated $MoS_2$ Nanosheets Using Spectroscopic Probes. Journal of Physical Chemistry C **2019**, 123, 27264–27271.

4. Layer-controlled large area $MoS_2$ layers grown on mica substrate for surface-enhanced Raman scattering. Applied Surface Science **2015**, 357, 1708–1713.

5. Kim, Y.; Jhon, Y.; Park, J.; Kim, C.; Lee, S.; Jhon, Y. Plasma functionalization for cyclic transition between neutral and charged excitons in monolayer $MoS_2$. Scientific Reports 6.

6. Van Bremen, R.; Vonk, K.; Zandvliet, H. J.; Bampoulis, P. Environmentally Controlled Charge Carrier Injection Mechanisms of Metal/$WS_2$ Junctions. Journal of Physical Chemistry Letters **2019**, 10, 2578–2584.
